# Evaluation of Sensible Heat Flux and Evapotranspiration Estimates Using a Surface Layer Scintillometer and a Large Weighing Lysimeter

**DOI:** 10.3390/s17102350

**Published:** 2017-10-14

**Authors:** Jerry E. Moorhead, Gary W. Marek, Paul D. Colaizzi, Prasanna H. Gowda, Steven R. Evett, David K. Brauer, Thomas H. Marek, Dana O. Porter

**Affiliations:** 1 USDA-ARS Conservation and Production Research Laboratory, PO Drawer 10, Bushland, TX 79012, USA; gary.marek@ars.usda.gov (G.W.M.); paul.colaizzi@ars.usda.gov (P.D.C.); steve.evett@ars.usda.gov (S.R.E.); david.brauer@ars.usda.gov (D.K.B.); 2 USDA-ARS Grazinglands Research Laboratory, 7207 West Cheyenne St., El Reno, OK 73036, USA; prasanna.gowda@ars.usda.gov; 3 Texas A&M AgriLife Research, 6500 Amarillo Blvd W, Amarillo, TX 79106, USA; tmarek@ag.tamu.edu; 4 Texas A&M AgriLife Extension Service, 1102 E FM 1294, Lubbock, TX 79403, USA; dporter@ag.tamu.edu

**Keywords:** irrigation, energy balance, water management, semi-arid regions

## Abstract

Accurate estimates of actual crop evapotranspiration (ET) are important for optimal irrigation water management, especially in arid and semi-arid regions. Common ET sensing methods include Bowen Ratio, Eddy Covariance (EC), and scintillometers. Large weighing lysimeters are considered the ultimate standard for measurement of ET, however, they are expensive to install and maintain. Although EC and scintillometers are less costly and relatively portable, EC has known energy balance closure discrepancies. Previous scintillometer studies used EC for ground-truthing, but no studies considered weighing lysimeters. In this study, a Surface Layer Scintillometer (SLS) was evaluated for accuracy in determining ET as well as sensible and latent heat fluxes, as compared to a large weighing lysimeter in Bushland, TX. The SLS was installed over irrigated grain sorghum (*Sorghum bicolor* (L.) Moench) for the period 29 July–17 August 2015 and over grain corn (*Zea mays* L.) for the period 23 June–2 October 2016. Results showed poor correlation for sensible heat flux, but much better correlation with ET, with r^2^ values of 0.83 and 0.87 for hourly and daily ET, respectively. The accuracy of the SLS was comparable to other ET sensing instruments with an RMSE of 0.13 mm·h^−1^ (31%) for hourly ET; however, summing hourly values to a daily time step reduced the ET error to 14% (0.75 mm·d^−1^). This level of accuracy indicates that potential exists for the SLS to be used in some water management applications. As few studies have been conducted to evaluate the SLS for ET estimation, or in combination with lysimetric data, further evaluations would be beneficial to investigate the applicability of the SLS in water resources management.

## 1. Introduction

In arid and semi-arid regions of the world such as the Texas High Plains, finite groundwater resources are being mined, often with little to no surface water available as an alternate irrigation source. In the Texas High Plains, irrigation pumping for agricultural crop production accounts for the overwhelming majority of total groundwater withdrawals [1]. Effective irrigation management is essential for extending the longevity of limited water resources in these intensively irrigated production areas. Most effective irrigation management (scheduling) strategies rely on accurate estimates of evapotranspiration (ET) to account for crop water use and evaporative water losses. ET is a term that represents water lost through evaporation from the soil or plant surface and water used through plant transpiration. In the Texas High Plains, ET is the largest water loss component in the soil water budget [1,2]; hence, accurate ET estimates are vital for determining crop water demand and managing irrigation. Groundwater recharge in the Texas High Plains, and the surrounding Southern Ogallala Aquifer region can be as low as ~11 mm year^−1^ [3]. With such small recharge rates, the Ogallala Aquifer is essentially a finite water resource. Assuming an 11 mm recharge rate across all 5.4 million ha (13.4 million ac) in the northern half of the Texas panhandle (Texas Water Development Board Region A) would supply 0.60 km^3^ (484,000 ac-ft) of recharge to the aquifer. In contrast, 2010 agricultural water withdrawals were estimated to be 1.81 km^3^ (1.47 million ac-ft) for the same area [1]. Water conservation, in part through ET-based irrigation scheduling, is paramount to extending the longevity of this limited water resource for future generations.

Accurate measurement or estimation of ET can be difficult; however, numerous instruments and methods are available. One widely used method is the reference ET (ET_ref_) and crop coefficient product. Meteorological data are used to estimate ET_ref_, which corresponds to the water demand of a reference crop, either a short, clipped grass or alfalfa [4]. To obtain ET for a specific crop using reference ET, a crop coefficient (K_c_) is used to adjust reference ET to crop specific ET, or ET_c_; thus, ET_c_ = K_c_ × ET_ref_ [5]. For irrigation scheduling, ET_c_ is typically calculated at a daily time step. ET_c_ estimates the amount of water that would be used by that crop if there were no water limitation for crop growth. Actual (field-based) crop ET may be less than ET_c_ due to stresses from insufficient available water, nutrients, pests, etc. As such, actual ET would potentially be more useful than ET_ref_ or ET_c_ for irrigation management. An issue with using actual ET in irrigation scheduling is that ET can be very difficult to determine accurately. Where ET_ref_ can be calculated from weather parameters using a weather station with a reference surface, efforts to determine actual ET not only require more advanced (and expensive) instrumentation but still only result in an estimate of ET. Current technologies for determining ET estimates include lysimeters, Bowen Ratio, Eddy Covariance (EC), scintillometry, field water balance using soil water measurements, remote sensing models, and others.

The aforementioned methods can all be used to estimate ET; however there are disadvantages of each. With the soil water balance approach, drainage and runoff components can be difficult to accurately determine. Although they are commonly considered relatively small in arid and semiarid regions, they need to be accounted for to obtain the best accuracy. In addition, soil water measurements are valid for a small point in a field creating spatial representation concerns. Weighing lysimeters are the most accurate method of assessing ET [6], but they are very expensive to install, maintain, and operate. In addition, they require a high level of operational knowledge and data processing experience to obtain accurate and representative measurements. Large weighing lysimeters are typically considered research tools and are not practical for generalized irrigation scheduling. The Bowen Ratio method has been used to determine ET from the energy balance, but it is an indirect measurement and can have issues of instrument bias and data discontinuity when the Bowen Ratio approaches −1 [7]. EC is a method of estimating turbulent fluxes and ET, but is known to have significant energy balance closure errors [8,9,10]. Remote sensing models have been extensively used for ET mapping; however, they have been commonly evaluated using EC [11,12,13]. Some studies have evaluated remote sensing ET using lysimeters [14,15,16], though lysimeter data are not available in many regions. Other, simpler methods to achieve highly accurate ET data would greatly benefit future research. Scintillometers are another type of indirect measurement instrument that has been extensively used for surface atmospheric dynamics research, but they can also have large errors. EC and scintillometry are two of the more common turbulent flux and ET methods typically used. They are relatively inexpensive and easy to deploy and maintain; and provide spatially averaged data. Scintillometers measure contributions to fluxes over a fixed path length and EC can measure contributions to fluxes over a variable area influenced by wind movement. The spatial average nature of EC and scintillometers account for variation within the area of measurement and provide a degree of representativeness. The main issue with the EC method is the lack of accuracy, as reported throughout the literature. Although scintillometers are being used in atmospheric research [17], applications in irrigation management research are relatively new and few in number. A thorough evaluation of scintillometers is needed to determine if they are suitable to measure ET for the purposes of irrigation scheduling and developing crop coefficients. Scintillometers are commonly used with the energy balance equation to determine ET from scintillometer measurements.

### 1.1. Energy Balance

In many studies, scintillometers are used with the energy balance. The energy balance equation is:

(1) $$LE=R_{n}-H-G,$$

where *R_n_* is the net radiation, *LE* is the latent heat flux, *H* is the sensible heat flux, and *G* is the soil heat flux (all with units of W·m^−2^) [18,19]. The energy balance explains the dynamics for the dispersion of radiant energy from the sun to the land surface. Energy from the sun is either reflected or absorbed. The portion absorbed is the *R_n_*, where it is positive when energy moves toward the surface (plant canopy) and negative when it moves from the surface. The absorbed *R_n_* can then be distributed to the soil as *G*, to the air as *H*, or provide energy to evaporate water as *LE*. Sign conventions for Equation (1) vary, but in this study, *G* is positive when flux moves toward the soil surface, *LE* is positive when flux moves toward the plant canopy, and *H* is positive as flux moves from the canopy to the air.

### 1.2. Scintillometry

A scintillometer consists of a transmitter and receiver, separated by a specified path length. Scintillometry, as applied to agricultural and other landscapes, uses a beam of electromagnetic radiation of known wavelength transmitted across a relatively large distance (100 m–4.5 km). The beam intensity fluctuates due to absorption and diffraction as it encounters eddies in the air. These fluctuations, or scintillations, can be used to determine the structural parameter of the refractive index of air, which can be used to calculate the structural parameters for temperature and humidity, and *H*. The calculations to obtain *H* from scintillometers are based on the Monin–Obukhov Similarity Theory (MOST). Details of MOST can be found in Arya [20], Foken [21], Hartogensis [22], McAneney et al. [23] and Monin et al. [24].

The main output of a scintillometer is the natural logarithm of the received beam intensity. Since the SLS uses two parallel beams, the calculation methodology is different. By using two beams, the covariance between the two can be calculated as:

(2) $$B_{12}=4\pi^{2}K^{2}\int_{0}^{L}\int_{0}^{\infty }k\emptyset_{n}\left(k\right)J_{0}\left(kd\right)sin^{2}\left[\frac{k^{2}\left(L-x\right)}{2KL}\right]\frac{4J_{1}^{2}\left(\frac{KDx}{2L}\right)}{{\left(\frac{kDx}{2L}\right)}^{2}}dkdx,$$

where *B*_12_ is the covariance between the beams, *K* is the wavenumber (*K* = 2*π*/*λ* rad m^−1^), *D* is the detector diameter (mm), *d* is the beam separation distance (mm), *L* is the path length (m), *x* is the coordinate along the path, *k* is the von Karman constant, *ϕ_n_* is the three dimensional spectrum of the refractive index inhomogeneities, and *J*_0_ and *J*_1_ are Bessel functions of the first kind [25]. The term *ϕ_n_* is calculated as:

(3) $$\emptyset_{n}\left(k\right)=0.33C_{n}^{2}k^{-\frac{11}{3}}f_{\emptyset }\left(kl_{0}\right),$$

where $C_{n}^{2}$ is the structural parameter of the refractive index of air (m^−2/3^) and *f_ϕ_*(*k*$l_{0}$) is the refractive index decay function. Equation (3) can be inserted into Equation (2) to define the covariance and the variance if $C_{n}^{2}$, the inner scale of turbulence ($l_{0}$), and the instrument physical dimensions are known, thus allowing the use of *B*_12_ and *B*_1_ or *B*_2_ to derive $C_{n}^{2}$ and $l_{0}$ [25]. Since the SLS measures variances in intensity, $\sigma_{i}^{2}$, rather than variances of log amplitude, *B_i_*, $\sigma_{i}^{2}$ must be converted to *B_i_*:

(4) $$B_{i}=\frac{1}{4}\log\left(1+\frac{\sigma_{i}^{2}}{{\langle I_{i}\rangle }^{2}}\right),$$

where *I_i_* is the intensity [25]. From the SLS, $C_{T}^{2}$ can be calculated from $C_{n}^{2}$ as:

(5) $$C_{T}^{2}=C_{n}^{2}T^{4}{\left(ap\right)}^{-2},$$

where *T* is the temperature in Kelvin, *p* is the atmospheric pressure in mbar, and *a* is a constant of 7.89 × 10^−5^ K/mbar at the 670 nm wavelength [25].

One advantage SLS offers over other point source measurements, is that the fluxes can be determined over shorter lengths and at heights closer to the surface [26]. Also, the SLS determines the $l_{0}$, which is proportional to the dissipation rate of the turbulent kinetic energy, *ε*, which are used to determine the friction velocity ($u_{*}$) and temperature scale ($T_{*}$). Sensible heat flux is calculated from $u_{*}$ and $T_{*}$ as:

(6) $$H=-\rho C_{p}u_{*}T_{*},$$

where $C_{p}$ is the specific heat of air (kJ·kg^−1^) and ρ is the air density (kg·m^−3^). The complete process to determine *H* from the SLS is provided in Savage [27] and Scintec [25].

Many different models of scintillometers are available, which differ based on their wavelength and aperture diameter. The large-aperture scintillometer (LAS) has a wavelength around 880 nm and an aperture of 10–30 cm. Microwave scintillometers (MWS) have an aperture around 30 cm and a wavelength of 1–3 mm. Several studies have evaluated scintillometry using the LAS and/or the MWS due to their long operational range. The MWS is noted to have the ability to measure LE directly [28,29,30] as the wavelength is sensitive to humidity fluctuations. Samain et al. [31] found RMSE values of approximately 14 W·m^−2^ for both LE and H between ETLook, TOPMODEL-based Land-Atmosphere Transfer Scheme (TOPLATS), and a LAS. Samain and Pauwels [32] found an RMSE of 0.1 mm·d^−1^ between an LAS and ET calculated from the Penman–Monteith equation. Meijninger et al. [33] found LE from a single LAS and a dual LAS–MWS setup were within 25% of the LE obtained from EC. Guyot et al. [34] found good agreement from a LAS as compared to EC and the water balance approach. Yee et al. [35] found a standalone LAS to be more suitable than the two-wavelength approach or a standalone MWS for a semi-arid environment. Studies evaluating a LAS or validating using instruments other than a lysimeter are abundant in the literature [28,36,37,38,39,40,41,42,43].

The SLS has been available for many years; however, SLS agricultural and crop-based studies are not abundant in the literature. Research related to the LAS or MWS vastly outweighs research using the SLS. Most SLS literature is related to atmospheric research and the number of publications is still small compared to other instruments. A few studies illustrate the benefits of the SLS over other flux measurement instruments, such as EC. Odhiambo and Savage [44] provided benefits and disadvantages of SLS, as well as an overview of scintillometry and various scintillometers. They note that the operation of a scintillometer using a path average approach provides the benefit of allowing for shorter time intervals. In addition, scintillometry does not require corrections (such as frequency response corrections for EC), that are generally subjective, to be applied. Lastly, Odhiambo and Savage [44] mentioned that since the variance of the logarithm of beam amplitude is the measurement used, any calibration multipliers and constants cancel or are removed by band-pass filtering. The disadvantages given by Odhiambo and Savage [44] include using MOST, which requires the effective beam height and zero-plane displacement height. In addition, the scintillometer itself cannot determine the direction of H, so the temperature difference from additional temperature sensors or other methods must be used to determine the flux direction.

Savage et al. [45] investigated H using a SLS, in addition to EC and Bowen Ratio, over natural grassland over a longer time period. They noted that most SLS studies were rather short, commonly with a time frame of a few days to a few weeks. Therefore, they performed an analysis using more than 30 months of data. They used averaging periods of two minutes for energy balance components, ET_ref_ estimates, and SLS measurements, 20 min for Bowen Ratio, and 30 min for EC. Their regression analysis of daily ET between Bowen Ratio to SLS (BR-SLS) showed an r^2^ of 0.87, while a similar analysis of EC to SLS (EC-SLS) showed improved correlation with an r^2^ of 0.96. The ET estimate comparison showed an RMSE of 0.58 mm·d^−1^ for the SLS-BR and 0.31 mm·d^−1^ for SLS-EC. Comparisons to ET_ref_ were not made.

De Bruin et al. [46] also found good correlation between H from an EC system and SLS near Uppsala, Sweden over a mixed landscape encompassing mostly agricultural land. Their regression analysis showed the flux from the EC was 1% greater and the flux comparison between EC and SLS had an r^2^ of 0.95. They also compared $u_{*}$ from the SLS and EC and found an r^2^ of 0.87.

Nakaya et al. [47] compared an SLS with an EC system over a deciduous forest in Japan. They found the SLS to overestimate H and the LE, as compared to their EC system. Since EC systems often underestimate LE, the SLS values could have been closer to the real LE. They also observed that the SLS underestimated $u_{*}$, especially when the values of $u_{*}$ were large. They concluded that the cause in the underestimation of $u_{*}$ is due to systematic errors in the determination of *l*_0_.

Hartogensis et al. [48] analyzed data from a SLS using data from the Co-operative Atmosphere Surface Exchange Study (CASES-99) experiment conducted in Kansas. They noted the benefits associated with using an SLS over EC, including a shorter averaging interval, shorter measurement height, and shorter path length, while also noting some disadvantages to the SLS. They noted that conversion from *l*_0_ and $C_{n}^{2}$ to H is done empirically using MOST. This can result in a bias in *l*_0_, causing an overestimation of $u_{*}$ for small $u_{*}$ values and underestimation of $u_{*}$ for large $u_{*}$ values [46]. The CASES-99 experiment deployed both a SLS and an EC system. Hartogensis, De Bruin and Van de Wiel [48] found that from the shorter averaging interval from the SLS (6 s for CASES-99), intermittent turbulence could be detected, whereas the longer averaging interval for the EC (30 min) would mask some of the intermittent turbulence. Comparing the *ε*, they showed the bias in the *l*_0_ caused a bias in the SLS *ε* where an overestimation of *ε* occurred for small values of *ε* and an underestimation occurred for large values of *ε*. Comparing *ε* obtained from SLS and EC showed good agreement between the two instruments. Comparing $C_{T}^{2}$ obtained from SLS and EC showed less agreement where the SLS $C_{T}^{2}$ was consistently larger [48]. In their analysis of H, they found the SLS to overestimate the EC H for fluxes greater than −50 W·m^−2^. They concluded that most of the error in the SLS data, as compared to EC, can be improved upon by taking the aperture diameter as 2.6 mm instead of the manufacturer reported 2.7 mm. This was tested in response to a finding in De Bruin et al. [46] where empirical functions were proposed to account for noise and inactive turbulence (where turbulence does not create mixing).

The previously described studies reiterate that evaluation of the SLS has used EC as validation. SLS studies involving lysimeter comparisons were not found in the literature and leave an opportunity for evaluation against highly accurate data. In addition, the SLS has not been widely evaluated for determining ET. In this study, an SLS was evaluated against data from a large weighing lysimeter for determination of hourly and daily H and ET for agricultural crops.

## 2. Materials and Methods

### 2.1. Site Description

**Figure 1:**
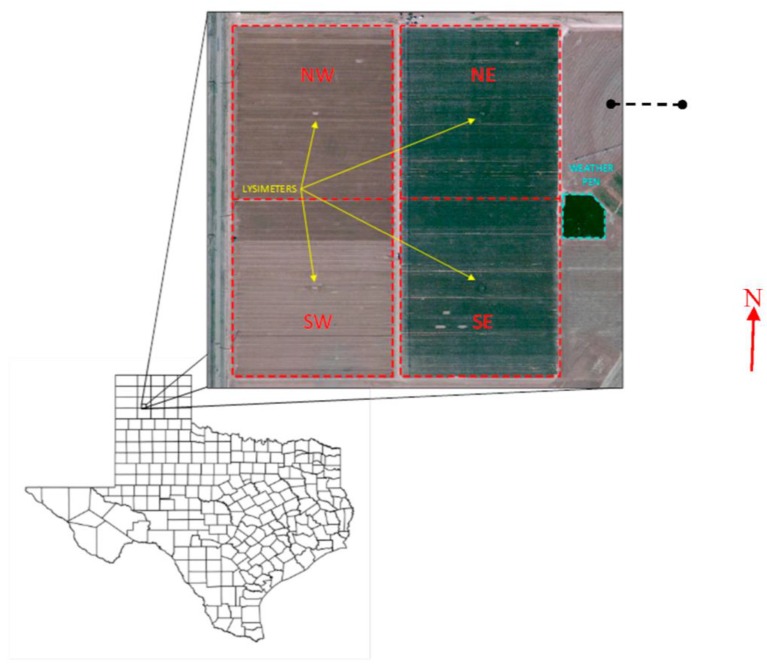
The study location with placement of large weighing lysimeters at the centers of four square fields at the USDA-ARS Conservation and Production Research Laboratory, Bushland, TX. The research weather pen is shown adjacent to the east side of the east lysimeter fields. The dashed black line in the NE field illustrates the path of the scintillometer radiation.

This study was conducted at the USDA-ARS Conservation and Production Research Laboratory, located 17 km west of Amarillo, TX. The region is classified as semi-arid with approximately 450 mm (17.7 in.) average annual precipitation. The study site is located inside a 19-ha square-shaped field, which is split into four, 4.7 ha quadrants (see Figure 1). Each quadrant is roughly 200 m by 220 m and contains a large weighing lysimeter located in the center of the quadrant. The east quadrants are irrigated with a subsurface drip irrigation system, and the west quadrants are irrigated using a low pressure lateral move sprinkler irrigation system. The NE quadrant was used for this study as it provides the greatest fetch with respect to the predominant wind direction (south southwest). In the 2015 summer growing season, the field was planted with grain sorghum (*Sorghum bicolor* (L.) Moench) at a rate of 210,000 seeds per ha (85,000 seeds per acre), which was fully irrigated using subsurface drip lines buried at 30 cm (12 in.) depth and 152 cm (60 in.) lateral spacing, centered in alternate interrows. In the 2016 growing season, the field was planted with grain corn (*Zea mays* L.) at a rate of 104,000 seeds per ha (42,100 seeds per acre), which was also fully irrigated using the previously described subsurface drip irrigation system.

### 2.2. Lysimeter Description

Hourly H and ET derived from SLS data (SLS-20, Scintec AG, Rottenburg, Germany) were evaluated by comparing against lysimeter data. Estimates from the SLS are denoted “sls” subscript and measurements by the lysimeter are denoted by “lys” subscript. The large weighing lysimeter measures 3 m by 3 m on the surface by 2.3 m deep over a fine sand drainage base. It contains an undisturbed monolith of Pullman silty clay loam soil. The soil container rests on a large balance scale equipped with a counterbalance and load cell system. Initial design and installation details of the lysimeter were provided by Marek et al. [49] and Schneider et al. [50]. The lysimeter was later equipped with drainage effluent tanks suspended from the lysimeter by load cells for separate measurement of drainage mass without changing total lysimeter mass. Load cell output is measured and recorded by a precision datalogger (CR7 in 2016, upgraded to CR6 for 2016, Campbell Scientific, Logan, UT, USA). Load cell voltage outputs are converted to mass using calibration equations, and five-minute means are used to develop a base dataset for subsequent processing [51]. Lysimeter mass in kg is converted to a mass-equivalent relative lysimeter storage value (mm of water) by dividing the mass by the relevant surface area of the lysimeter (~9 m^2^) and the density of water (1000 kg·m^−3^). See Evett et al. [6] for a thorough description of the calculations, and of the lysimeter operation, sensors, and ancillary equipment. Equivalent mass values allow for changes in lysimeter mass to be expressed in terms of water flux, defined as mm of water lost or gained per unit time. The lysimeter datalogger mass resolution is better than 0.001 mm when converted to equivalent depth of water. Lysimeter accuracy is, however, determined by the RMSE of calibration, which ranged from 0.01 mm to 0.05 mm [6,51]. Lysimetric data quality assurance and quality control (QA/QC) and data processing techniques were discussed by Marek et al. [52].

In a few instances, field operations prohibited accurate measurements with the lysimeter. For example, maintenance on the lysimeter, or instruments mounted near the lysimeter, required personnel to step onto the lysimeter container, temporarily increasing the mass. Other operations include draining the percolation storage tanks, which causes an overall decrease in the mass, irrigation applications, and foot traffic associated with taking neutron probe soil water measurements. The amount of water drained is measured, but data recorded during the process of draining the tanks is not valid and usable. For sub-daily data, periods of precipitation or irrigation reduce data availability since the precipitation/irrigation cannot be accounted for in the ET_lys_ for short periods, such as hourly or 30 min intervals. In the instance where operations, precipitation, or irrigation limited accuracy of collected data, the data from those time intervals were omitted. Precipitation and irrigation events were the dominant reason for data exclusion in this study. In the instances where lysimeter data were omitted, the corresponding SLS data also were excluded. For daily and longer time steps, the data can be evaluated and/or corrected to account for periods when hourly data are not available. This involves subtracting the lysimeter mass value at midnight from the mass at midnight of the previous day. With this process, temporary changes in mass, such as taking neutron probe readings, can be disregarded. Permanent changes in mass such as irrigation, precipitation, and emptying drainage tanks can be accounted for by adding/subtracting the mass of water added or removed to the midnight-midnight mass difference. Details on the evaluation and correction of daily lysimeter data can be found in Marek et al. [52].

### 2.3. Scintillometer Installation

The SLS was installed in the NE lysimeter field with an east-west path length of 100 m passing approximately 25 m north of the lysimeter (Figure 1). The measurement height was 1.73 m in 2015 and 2.84 m in 2016. The maximum crop heights were 1.3 m and 2.3 m for grain sorghum in 2015 and corn in 2016, respectively. The instrument height of 1.73 m was the highest the SLS could be raised with the tripod supplied with the instrument. To compensate for the taller corn crop, a taller tripod was fabricated. The 2.84 m height in 2016 was the tallest the fabricated tripod could achieve. The SLS was connected to a custom built PC running SLS configuration and processing software (SRun version 1.28, Scintec AG, Rottenburg, Germany). The SLS was oriented with the transmitter facing east so the path was as close as possible to perpendicular to the predominant wind direction. The SLS measurements for the period of 29 July–22 August 2015 and 23 June–2 October 2016 were used for this study. Sensible heat flux was determined using SRun software, and H_sls_ data were provided for assumptions according to stable (H_stable_) and unstable (H_unstable_) atmospheric conditions. Using scintillometry, the value of H can be determined, but not the direction of the flux [44]. The flux direction must be determined during data post-processing using supplemental instrument data. In stable atmospheric conditions, the H will be negative while H will be positive in unstable conditions. Several methods are reported in the literature for determining flux direction [36,44,53,54]. One is based on the view that the atmosphere is typically stable at night and becomes unstable during the day [41]. To account for this pattern, assuming H_stable_ for nighttime hours and H_unstable_ for daytime can be used to assign H direction (H_sun_). Another method of determining flux direction (H_dT_) is to use the temperature gradient as determined from two co-located air temperature sensors at different heights [43]. The temperature gradient provides an indication of the stability of the atmosphere near the surface, as well as an indication as to the direction of sensible heat flux since sensible heat will flow across the gradient from high to low. In stable atmospheric conditions, the gradient indicates air temperature increases with height. This will cause heat energy to flow down into the crop canopy and indicate negative H. In an unstable atmosphere, temperature decreases with height, so heat will move vertically and away from the canopy, resulting in positive H. In 2015, air temperature was measured at 1.2 and 2.8 m, while in 2016 air temperature was measured at 2.8 and 6.4 m. The air temperature measurement heights provided a measurement at or below and above the SLS beam for both years. Both of the described methods were used to determine ET_sls_ and compared to the lysimeter to determine the effects of H_sls_ direction methodology. For H_sun_, the daytime and nighttime hours were determined using sunrise and sunset times for the study period (7:00 a.m. and 9:00 p.m. CDT, on average, respectively).

**Figure 2:**
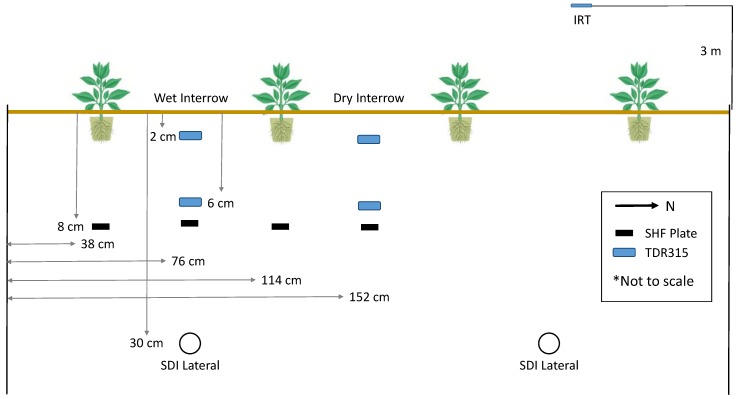
Soil sensor placement within the lysimeter. Shown are relative placement of soil heat flux (SHF) plates, model TDR315 soil water content sensors, and subsurface drip irrigation lines. The TDR315 sensors give accurate soil temperature readings, eliminating the need for thermocouples where they are used.

Additional instruments included air temperature and relative humidity sensors (HMP155, Vaisala, Helsinki, Finland), four soil heat flux plates (HFT-3, Radiation Energy Balance Systems, Bellevue, Washington, DC, USA), four soil water sensors (TDR315, Acclima, Meridian, ID, USA), an infrared thermometer (IRT), and a net radiometer (Q*7, Radiation Energy Balance Systems). The layout of the soil sensors within the lysimeter is presented in Figure 2. The instruments were connected to a datalogger (CR6, Campbell Scientific) with a measurement frequency of six seconds and averaged to an hourly interval.

The soil water and temperature data were used to calculate G at the soil surface by the calorimetric method, and surface G was used in all energy balance calculations. The calorimetric method used in this study was described by Colaizzi et al. [55]; briefly, it used the soil water and temperature measurements to calculate the change in soil heat storage between the surface and the depth of the soil heat flux plates in 1 h time steps. Using measured R_n_ and surface G provided the available energy to accompany the H measurement and LE calculation. Data from the R_n_ and surface G instruments on the lysimeter were used with the SLS data.

H was back-calculated from the lysimeter by converting the ET_lys_ to *LE*, summing *LE* with surface soil heat flux and measured net radiation, and treating the residual of this energy balance to H. LE was calculated from ET by:

(7) $$LE=\frac{\mathrm{ET}\left(\mathrm{mm}\right)\lambda }{time\left(\sec\right)},$$

where *λ* is the latent heat of vaporization (J·kg^−1^). The latent heat of vaporization was calculated from the surface temperature (*T_s_* °C) by:

(8) $$\lambda =\left(2.501-0.0236T_{s}\right)\times 10^{6}.$$

A south-facing, nadir IRT installed 3 m above the lysimeter was used to determine the surface temperature, which was then used to calculate the latent heat of vaporization for each hourly period. Using the ET_lys_ data, the LE was calculated by multiplying the ET_lys_ by the latent heat of vaporization and dividing by 3600 s to convert to the hourly time period.

During nighttime hours, *LE*, and subsequently ET, become very small [56]. The much smaller values can exhibit much more relative variation; even though the magnitude of the differences may be small, relative percentage differences can be large. To determine the effects of including nighttime ET_sls_, evaluations were also conducted using only daytime ET_sls_ data. This analysis provided an indication as to how much of the overall variation was influenced by the much smaller nighttime values.

As most water management practices and modeling efforts use a daily time step, the 24 hourly periods were summed to provide an evaluation for daily ET_sls_ data. The 24-h summation was performed for both ET_lys_ and ET_sls_. Since some hourly intervals were omitted due to operations on the lysimeter, daily lysimeter data (midnight–midnight mass change) as determined by the processes outlined in Marek et al. [52] were also used to evaluate the 24-h summations from the SLS. The statistics of root mean square error (RMSE), percent RMSE (%RMSE), and regression analysis (H_sls_ regressed to H_lys_, ET_sls_ regressed to ET_lys_) were used as the basis of evaluation. The %RMSE provides a relational value that allows for comparison of results of different magnitudes. The %RMSE was calculated by dividing the RMSE by the average of the measured lysimeter data.

## 3. Results

**Figure 3:**
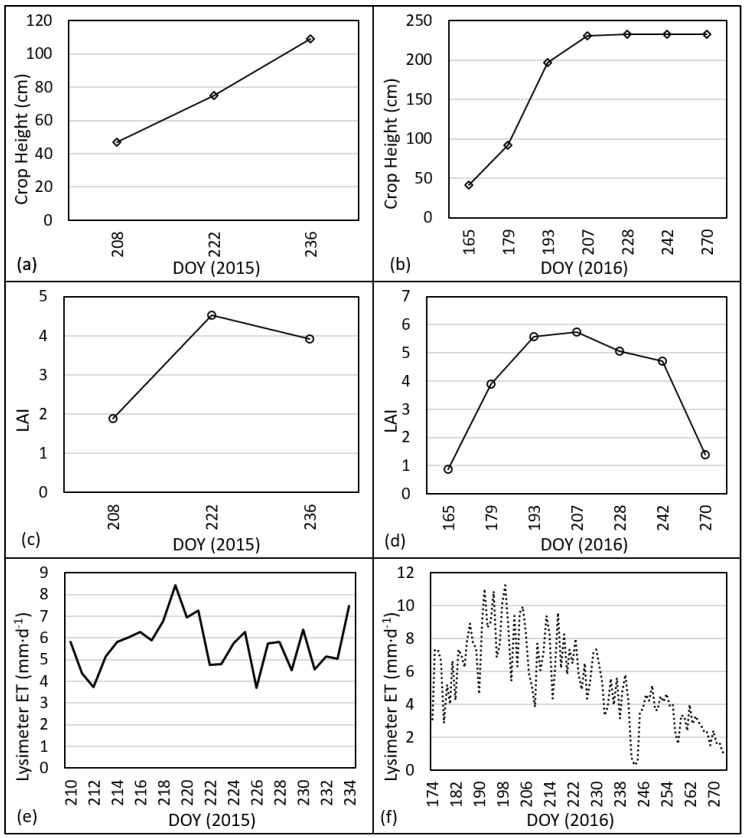
Crop height for 2015 (**a**) and 2016 (**b**), LAI for 2015 (**c**) and 2016 (**d**), and daily lysimeter ET for the 2015 (**e**) and 2016 (**f**) study periods.

Daily ET_lys_ values for 2015 and 2016, as well as crop height and leaf area index (LAI) are presented in Figure 3. An exceptionally wet year occurred in 2015 for the study location, which may have caused greater evaporation and thus higher ET_lys_ values as compared to 2016 for the same date range. Since the lysimeter field is irrigated using subsurface drip, the soil surface is typically dry and evaporation is minimized. When a precipitation event occurs, the soil and plant surfaces become wet and experience evaporation that does not occur with irrigation as the irrigation water typically does not reach the soil surface. In addition, during the data period for 2015, the sorghum crop was still in a vegetative growth stage with higher relative ET. The data for 2016 encompasses vegetative growth of the corn crop, but also, late season reproductive growth and grain filling. During the corn grain fill stage, ET_lys_ is lower and possibly reduced the overall hourly average values. Both years exhibit the pattern of lower ET_lys_ during the night hours, increasing from sunrise to a maximum around 14:00 and then decreasing. Although ET_lys_ is drastically reduced during the night, the measured ET_lys_ values illustrate that a small amount of ET does occur at night, which is not negligible [56].

Energy balance closure was 87% (based on the slope of the regression equation) when the available energy (AE: *R_n_*-*G*) was regressed against H_dT_ + LE_lys_. The energy balance closure error was similar to the %RMSE for H_dt_ (87% and 83%, respectively. This similarity leads to the inference that the energy balance closure error results from error in H_dt_ as compared to H_lys_. The results of the error analyses for the turbulent fluxes and ET_sls_ are presented in Table 1. Even though the H_sls_ had weak correlation with the H_lys_, and a large error, the resulting ET_sls_ had a much smaller error, especially for daily ET_sls_. This indicates that errors in determining H do not have a drastic impact on ET_sls_. In irrigated agriculture, H is commonly small, especially compared to LE. Large errors in a small component may not result in large error in the final product. Results for the AE and incoming solar irradiance regressed against ET_lys_ are also included in Table 1. This analysis showed the extent of error associated with estimating ET without measuring H. The slopes for the regression equations from AE and irradiance are provided in Table 1; however, the slope values are much larger than for ET and H since AE and irradiance values are considerably larger than ET. Errors for ET were larger using AE and irradiance as predictors of ET, which indicates that although large errors exist for H_sls_, ET_sls_ errors are still lower when accounting for the H component. The error rates and small error suggest that potential exists for additional research and potential improvements in SLS measurements for H, and possibly the resulting ET_sls_.

**Figure 4:**
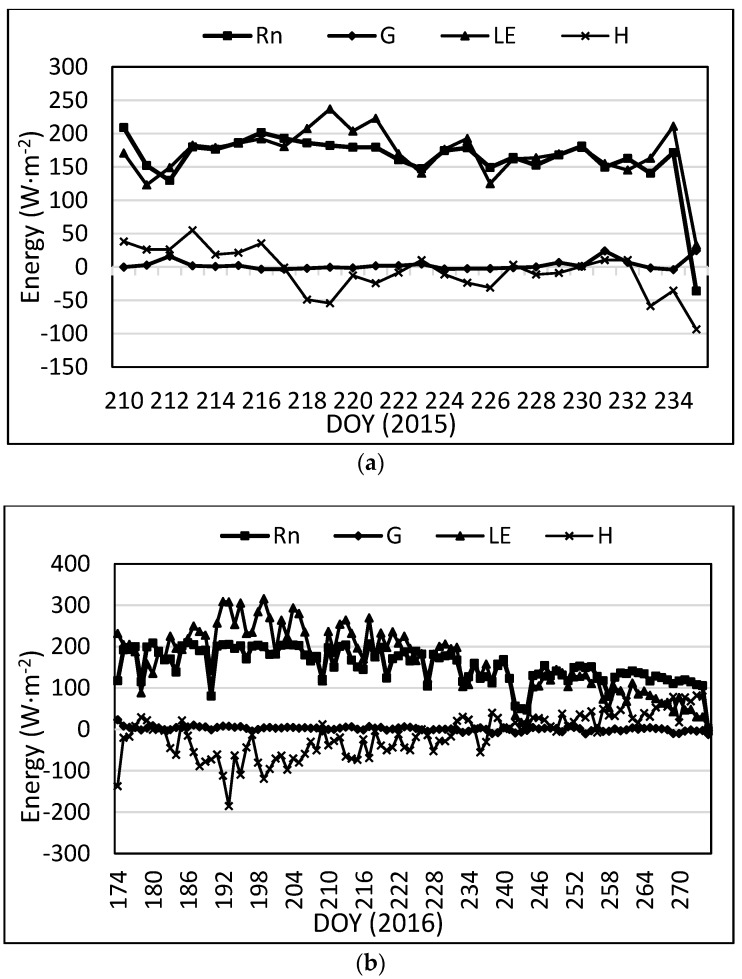
Average daily energy balance components on the lysimeter for (**a**) 2015; (**b**) 2016.

The average daily energy balance components for the lysimeter are presented in Figure 4. From the figure, *R_n_* and LE had the largest values, and H and *G* had much lower values. Since *R_n_* is the main energy input, when one component (LE) increases, another component (H) should decrease. This is not always the case as the environment in the Texas High Plains commonly generates advection where heat energy is transferred from a warmer, typically drier adjacent field to cooler irrigated fields. Evidence of advection is also seen in cases where *LE* exceeds *R_n_* [7,57]. The influx of heat energy from nearby (warmer) fields increases the energy available for evaporation of water during transpiration, thus increasing the latent heat flux.

**Figure 5:**
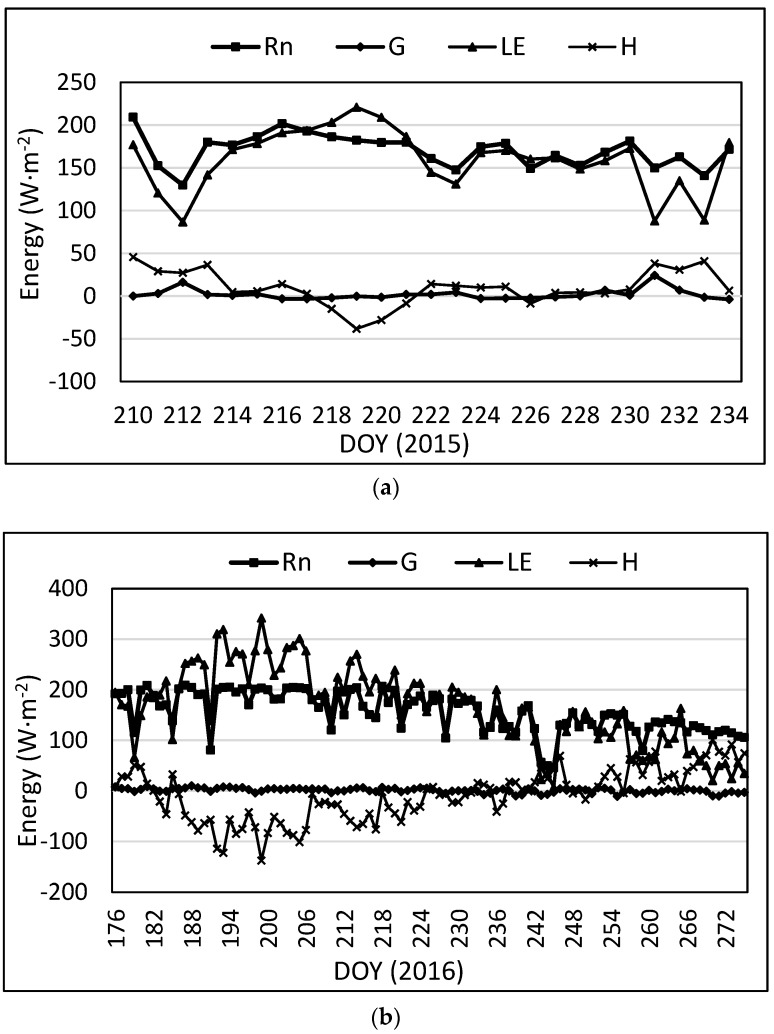
Average daily energy balance components for the SLS for (**a**) 2015; (**b**) 2016.

Figure 5 presents average daily data for all four components of the energy balance equation as determined by the SLS. For the SLS, the temperature difference was used to determine flux direction for H shown in Figure 5. In some instances, *LE* is greater than *R_n_* during the daytime, which in this case, corresponds to instances where LE was greater than *R_n_* with the lysimeter. This usually indicates the occurrence of advection rather than an *LE* error since the same higher LE was measured on the lysimeter for the same dates.

**Figure 6:**
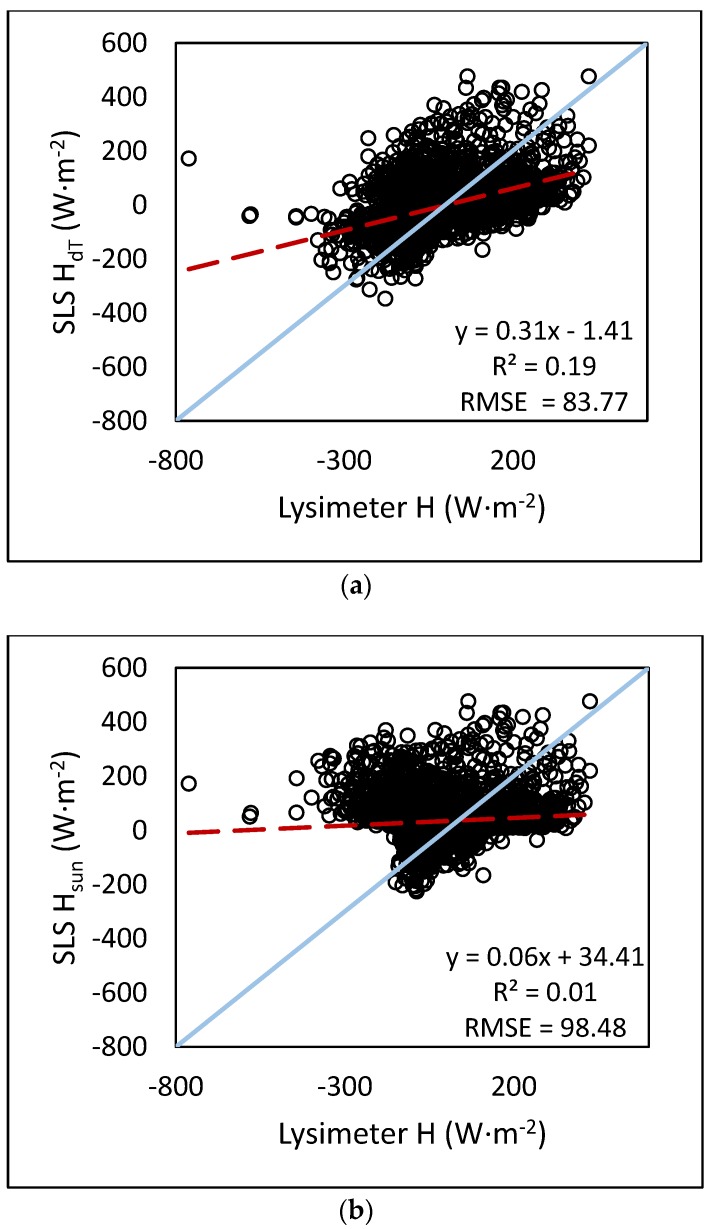
Comparison of hourly H_dT_ and H_sun_ from the SLS to lysimeter H for the combined 2015–2016 data based on lysimeter data regressed to (**a**) H_dT_; (**b**) H_sun_.

The results from the statistical analyses of H showed that using the temperature gradient produced a lower error than using H_sun_. The assumption that the atmosphere was stable at night and unstable during the day proved to not always be true. Comparisons of H_dT_ and H_sun_ are presented in Figure 6. The SLS overestimated H as compared to the lysimeter, which is illustrated by Figure 6 as well as the slope of the regression equation (0.31 and 0.06, respectively). H_dt_ from the SLS ranged from −347.7 to 475.8 W·m^−2^ with a mean of −5.1 W·m^−2^ and a standard deviation of 92.8 W·m^−2^. H_sun_ from the SLS ranged from −226.6 to 475.8 W·m^−2^ with a mean of 33.6 W·m^−2^ and a standard deviation of 93.7 W·m^−2^. H determined from the lysimeter ranged from −761.7 to 426.4 W·m^−2^ with a mean of −11.4 W·m^−2^ and a standard deviation of 130.1 W·m^−2^. The lysimeter showed a broader range of H values compared to the SLS, although mostly with regard to negative flux values. Although weak correlation is shown in Figure 6, the statistical analysis showed significant correlation (*p* < 0.05) for all data periods (2015 only, 2016 only, and 2015–2016) for H. The regression analyses showed all slopes were significant and all intercepts were significant (*p* < 0.05) with the exception of H_dT_ for the combined 2015–2016 dataset (*p* = 0.27). The measurement footprints of the lysimeter and SLS are not exactly the same. The lysimeter measures a 3 m by 3 m square whereas the SLS measures an average across the 100 m path, which is 25 m north of the lysimeter. Although care is taken to ensure the lysimeter is representative of the surrounding field, the difference in measurement footprint may contribute to the error in H.

**Figure 7:**
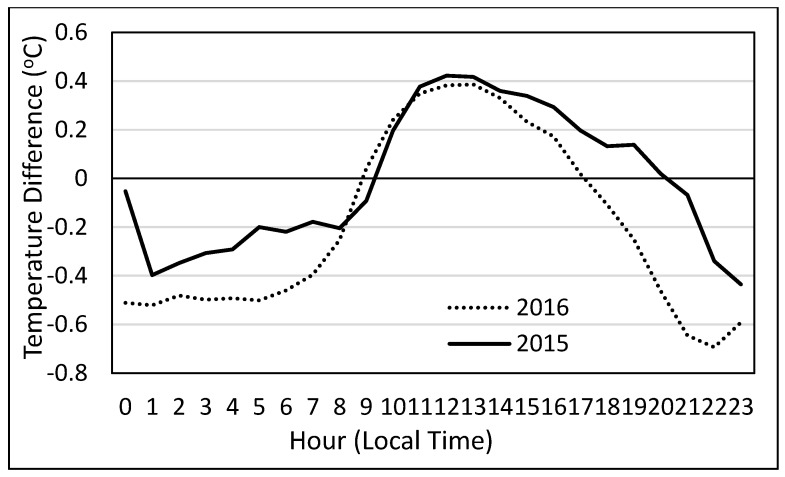
Temperature difference (calculated as the difference between values from two air temperature sensors at different heights—the lower sensor minus the higher sensor) and used as the indicator for flux direction.

Average hourly H directions, as indicated by the temperature gradient, for the study period are presented in Figure 7. A clear diurnal pattern is found with the flux direction, as it is common to find a stable atmosphere during nighttime hours and an unstable atmosphere during the daytime. Overall, stable conditions occurred more than unstable atmospheric conditions, with 31% and 42% of the hourly periods under unstable conditions for 2015 and 2016, respectively. Having a greater occurrence of stable atmospheric conditions was not expected as the atmosphere is typically unstable during the day, and the day length was longer than the length of nighttime period. Seeing more stable conditions than unstable indicates that stable atmospheric conditions must have occurred during some of the daytime hours, as also indicated by Figure 7. With 2015 being one of the wettest years on record for the study area, the relative humidity was greater for 2015 than 2016. The average relative humidity for the 2015 study period was 70% whereas the average humidity for 2016 was 59%. In addition, the average wind speed was lower for 2015 compared to 2016 (3.33 m·s^−1^ and 3.81 m·s^−1^, respectively). The lower wind speeds and greater humidity in 2015 may have allowed the atmosphere to stay more stable than in 2016. The greater humidity could have allowed the atmosphere to hold more heat and the lower wind speed could have reduced turbulence and atmospheric mixing.

**Figure 8:**
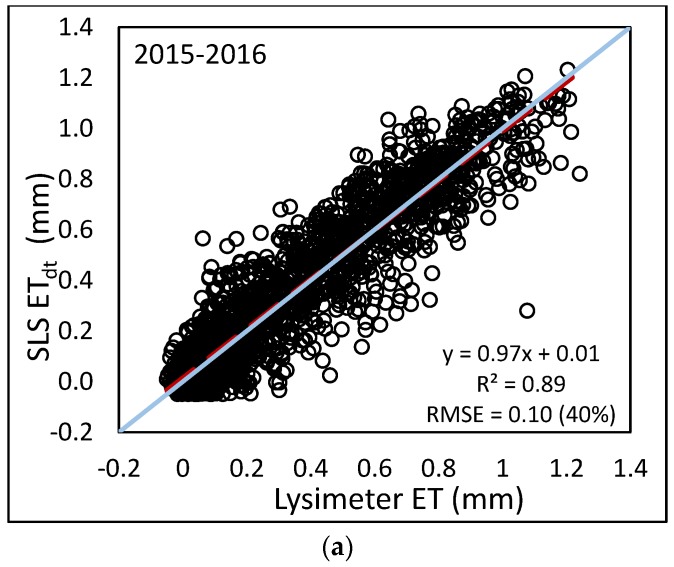

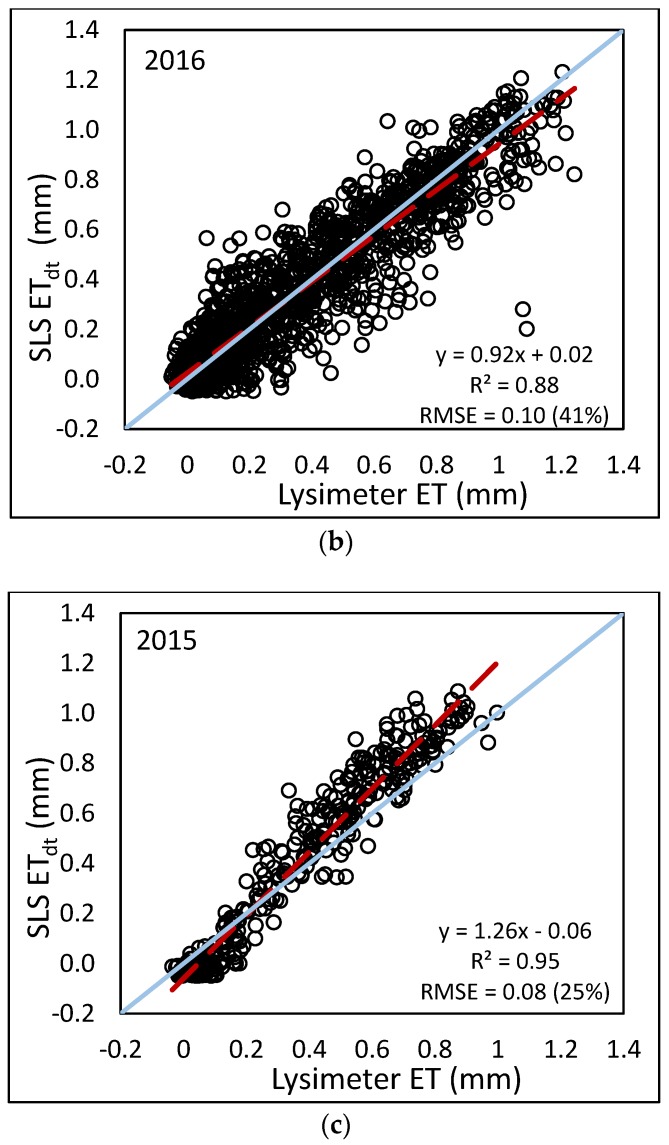
Comparison of hourly ET from SLS and lysimeter using (**a**) combined 2015$#x2013;2016 data; (**b**) 2016 only; (**c**) 2015 only.

In further analysis, hourly ET_sls_ was then calculated using the *LE* from the SLS and evaluated against lysimeter data. Hourly ET_sls_ correlated well with hourly ET_lys_ (see Figure 8). Coefficients of determination ranged from 0.88 to 0.95 with regression slopes ranging from 0.75 to 0.96. Although the slopes are close to 1, hourly ET_sls_ is slightly overestimated as compared to the lysimeter. RMSE values ranged from 0.08 to 0.10 mm·h^−1^, although the relative errors indicated by the %RMSE were much larger, ranging from 25 to 41%. The 2015 dataset resulted in smaller error than the 2016 dataset with %RMSE of 25% and 40%, respectively. This is possibly due to the timing of the 2015 data. The same period of 29 July–22 August for 2016 had a similar error to 2015 (0.09 mm, 30%). The period before 29 July, from 23 June to 28 July 2016 had an error of 0.11 (33%) and the period after 22 August from 23 August to 2 October 2016 had an error of 0.10 (72%). Although the RMSE values for the three periods in 2016 were similar, the %RMSE values are different. The sorghum crop in 2015 reached maximum plant height around 21 August, so the 2015 data were collected over at least a partially growing crop. The corn crop in 2016 reached maximum height around 15 July, so both later periods included the same crop height; however, the latter period was during grain fill when the corn ET values were less. For the period of 29 July–22 August 2016, average daily ET_lys_ was 6.73 mm; for the period of 23 August–2 October 2016, the average hourly ET_lys_ was 3.16 mm, roughly half of that for the middle period (see Figure 3a).

**Figure 9:**
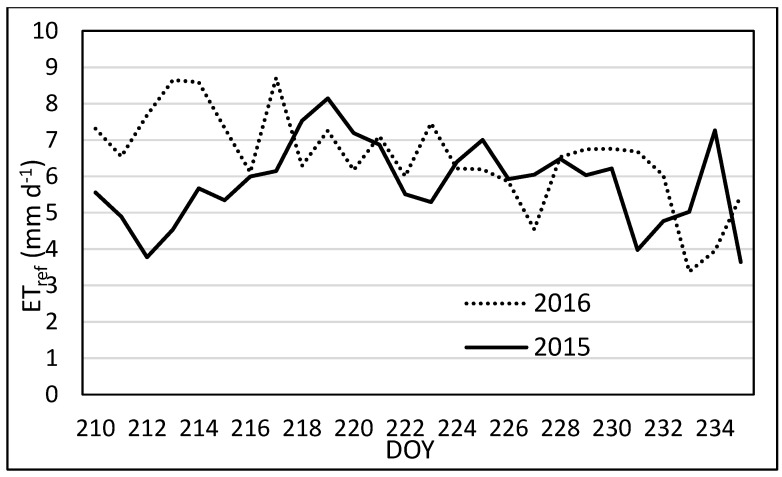
Daily ET_ref_ for the same DOY in 2015 and 2016, used as an indicator for differences in weather parameters.

In addition to the crop growth, the weather may have contributed the differences in accuracy of ET_sls_ for 2015 and 2016. Comparing ET_ref_ (which is calculated based on weather measurements) for the same date range of 29 July–22 August in 2015 and 2016, 2016 had larger values than 2015 (see Figure 9). Differences in ET_ref_ between the years were significant with a *p*-value of 0.04. Comparing maximum and minimum air temperatures as well as incoming solar radiation, 2016 showed much more variation in daily total incoming radiation. Incoming radiation data for 2015 were consistently higher and less variable than 2016, which may indicate more occurrences of clear skies in 2015. In addition to differences in incoming solar radiation, the difference between maximum and minimum air temperatures was greater in 2016 that in 2015. Although differences in temperatures were evident, statistical analysis showed no significant difference in maximum air temperature, but significant differences were present with minimum air temperature (*p*-value = 0.027). Wind speed measured at 2 m also showed significant differences (*p*-value = 0.030), with 2016 having higher wind speeds than 2015. The broader range of temperatures and higher wind speeds in 2016 may have had an adverse effect on SLS measurements.

**Figure 10:**
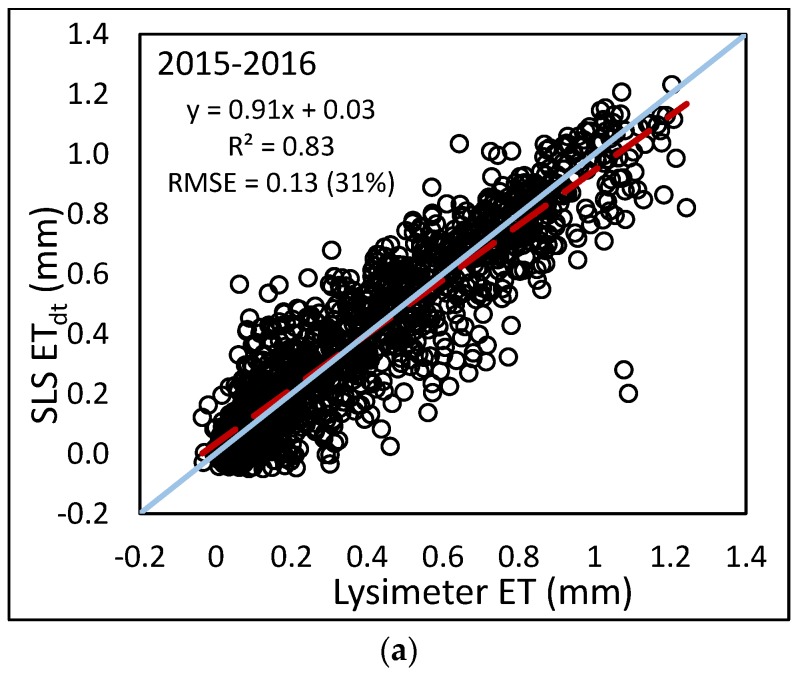

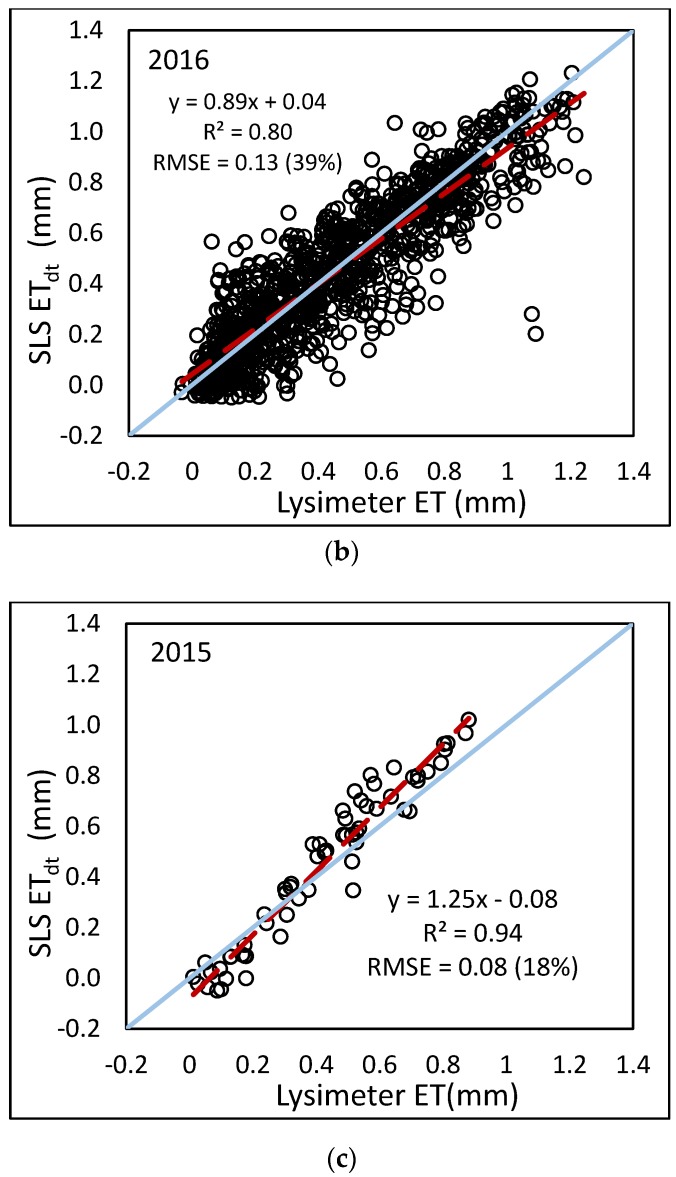
Comparison of daytime hourly ET from the SLS to lysimeter data using (**a**) 2015–2016 combined data; (**b**) 2016; (**c**) 2015.

In Figure 8, a cluster of values was found near zero for both the SLS and lysimeter. These small values likely occurred at night when photosynthesis dramatically slowed and less transpiration occurred. To investigate potential bias from the inclusion of these small values, the daytime ET_sls_ data were separated from the nighttime ET_sls_. The daytime ET_sls_ data were evaluated against daytime lysimeter measurements. Using only the daytime ET_sls_ resulted in a slight change in the regression equations, as well as increased variation, indicated by the reduced r^2^ values (see Figure 10). Although the variation increased, the RMSE decreased from 25 to 18%, 41 to 29%, and 40 to 31% for the combined 2015 only, 2016 only, and 2015–2016 data, respectively. The RMSE was larger using only daytime data; however the magnitude of ET_sls_ values were larger for the daytime, resulting in a smaller %RMSE.

**Figure 11:**
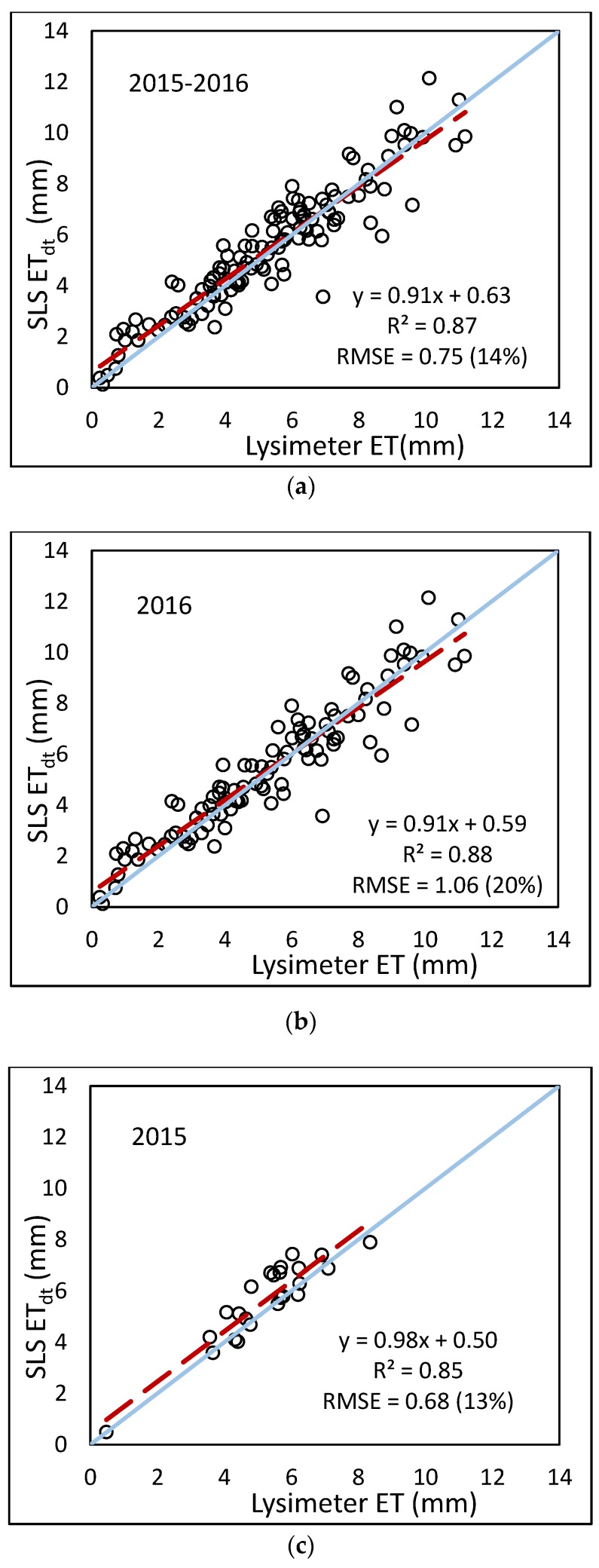
Comparison of summed daily ET from the SLS to summed hourly lysimeter data using (**a**) combined 2015–2016 data; (**b**) 2016 only; (**c**) 2015 only.

Regressions using the summed daily data (*n* = 24, 100, 124 for 2015 only, 2016 only, and 2015–2016, respectively) resulted in a slightly less correlation than the hourly regressions using hourly data. Coefficients of variation for the 2015 only, 2016 only, and combined 2015–2016 daily data were 0.85, 0.88, and 0.87, respectively, where the hourly data had r^2^ values of 0.95, 0.88, and 0.89. However, the slopes of the regressions were much closer to one for the daily sums, indicating that the regression equations were able to explain more of the variation (see Figure 11). All correlations were statistically significant with p-values less than 0.05. In addition, all slopes and intercepts (values presented in figures) were significantly different from one and zero, respectively. Regression on daily ET_sls_ had an RMSE of 0.68 mm·d^−1^ for the combined 2015–2016 data, which corresponded to less than half of the percent error for hourly ET_sls_ (14% compared to 31%). The reduction in error is likely due to the SLS having values greater and less than the lysimeter. At the hourly time step, these values contributed to the error; however, at the daily time step, the greater and lesser values canceled out after summation. It is important to note that the daily sums do not include all 24 data hours for all days. In the daily sums, the hourly data corresponding to missing data were omitted and only hours for which there were acceptable data were summed. Even though usable data for some days were not complete, the sums for both the SLS and the lysimeter still contained the same number of hourly values.

**Figure 12:**
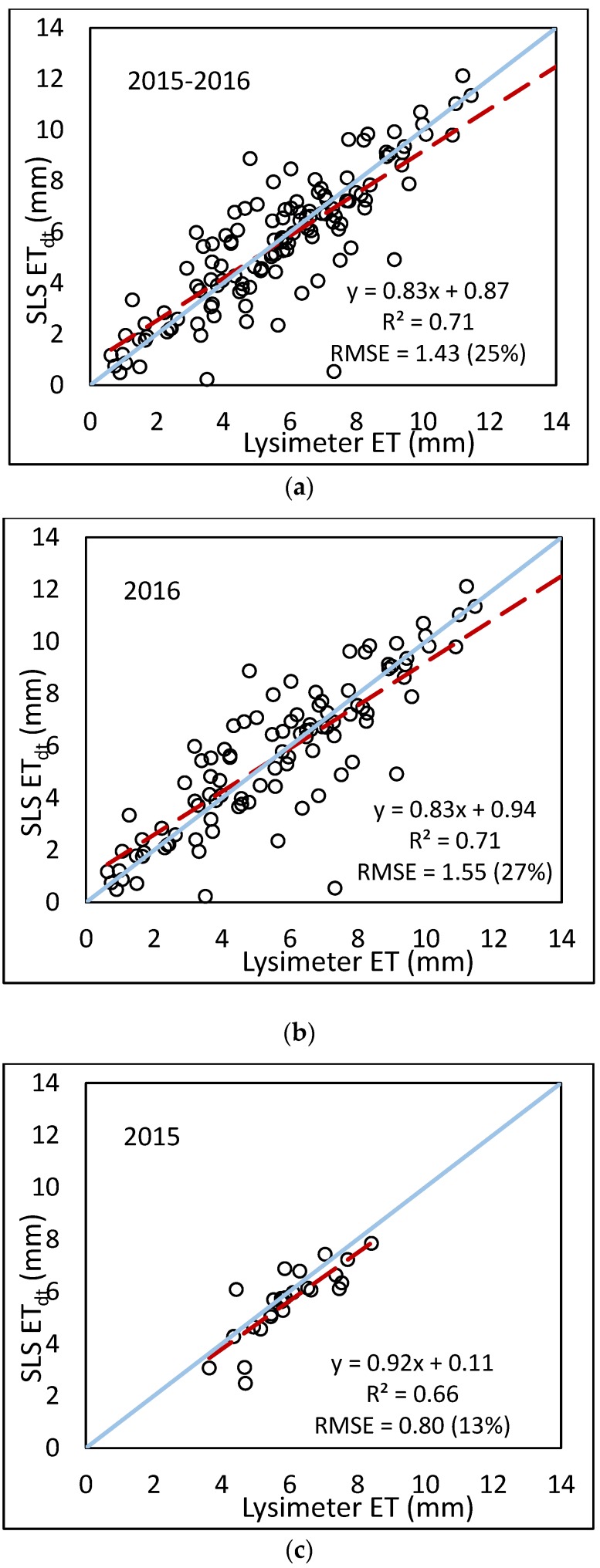
Comparison of summed daily ET from the SLS to daily (mass change from midnight to midnight) lysimeter data using (**a**) combined 2015–2016 data; (**b**) 2016 only; (**c**) 2015 only.

The results of the daily lysimeter (mass change from midnight to midnight) analysis are presented in Figure 12. For 2015, the results were similar to the summed hourly data; however there were some differences in the error for 2016 and the combined 2015–2016 data. In the hourly sum analysis, periods of precipitation or irrigation (the main reason lysimeter data were omitted) were not included in the analysis, therefore, the effects of the soil (or subsoil) wetting and drying were not considered. In the daily analysis, these periods were included, and the scintillometer may not have captured the change in soil and atmosphere dynamics caused by the soil wetting. With drip irrigation, the soil surface is typically dry and evaporation is minimized. With a rainfall event, the soil surface is wetted and evaporation increases thus reducing H and increasing *LE*. These transition periods, where energy shifts from H to *LE*, may not be adequately captured by the SLS.

## 4. Conclusions

A surface layer scintillometer (SLS) was installed in the subsurface drip-irrigated NE lysimeter field at the USDA-ARS-CPRL in Bushland, TX to evaluate accuracy of the SLS to determine sensible and latent heat fluxes. Summed daily ET from the SLS (ET_sls_) had as RMSE of 0.75 mm·d^−1^ as compared to the lysimeter with the regression slope (0.91) significantly different from one (*p*-value < 0.0001) and the intercept not significantly different from zero. Hourly ET regressed between the SLS and the lysimeter showed a slope of 0.91 and intercept of 0.03, both being significantly different from one and zero, respectively (*p*-values < 0.0001). Since the SLS cannot determine the direction of H, different post-processing techniques were evaluated. Using the difference between two air temperature sensors provides the temperature gradient and subsequent direction of H. The temperature gradient provided the best indication of the direction of H. Further analysis indicated that during the study period, stable atmospheric conditions were consistently present, which could have negatively affected the results from the SLS. Although the statistical performance of H_sls_ was not good, ET_sls_ correlated well, with r^2^ values greater than 0.80, and RMSE values of 0.13 to 0.75 mm for hourly and daily ET_sls_, respectively. For summed, daily ET_sls_, the error was reduced to roughly half the hourly error at 14%. The reduction in error was likely due to overestimations and underestimations canceling out upon summation. As mentioned in the introduction section, a need exists for portable instruments that can provide highly accurate ET data for validation data and other research purposes. The SLS presents the potential to fill this need. With error rates as low as 14%, the SLS exhibits potential for use in water management activities such as developing crop coefficients or irrigation scheduling when daily data can be used. However, for shorter time steps, error rates are larger and comparable to error rates from other instruments such as EC, and more research is needed to identify the causes of the errors in hourly data and potential improvements in accuracy of the method. Evaluating data with larger H values, such as dryland conditions, may provide more information regarding H discrepancies.

## Figures and Tables

**Table 1:** Regression statistics from comparison of hourly and daily H and ET data between SLS and lysimeter.

Data	RMSE	%RMSE	*R*^2^	Slope
H_dT_	83.8 W·m^−2^	83	0.19	0.31
ET (hourly)	0.10 mm	40	0.89	0.97
ET (daily)	0.75 mm	13	0.87	0.91
AE	0.16 mm	63	0.72	697
Irradiance	0.15 mm	62	0.72	920
